# Microchamber Cultures of Bladder Cancer: A Platform for Characterizing Drug Responsiveness and Resistance in PDX and Primary Cancer Cells

**DOI:** 10.1038/s41598-017-12543-9

**Published:** 2017-09-25

**Authors:** Pantea Gheibi, Shuxiong Zeng, Kyung Jin Son, Tam Vu, Ai-Hong Ma, Marc A. Dall’Era, Stanley Alexander Yap, Ralph W. de Vere White, Chong-Xian Pan, Alexander Revzin

**Affiliations:** 1 Department of Biomedical Engineering, University of California, Davis, CA 95616 USA; 2 Department of Internal Medicine, Division of Hematology/Oncology, University of California Davis, Sacramento, CA 95817 USA; 3 Division of Rheumatology, Allergy and Clinical Immunology, University of California, Davis, CA 95616 USA; 4 Department of Biochemistry and Molecular Medicine, University of California Davis, Sacramento, CA 95817 USA; 5 Department of Urology, University of California Davis, Davis, CA 95817 USA; 6 Department of Physiology and Biomedical Engineering, Mayo Clinic, Rochester, MN 55905 USA

## Abstract

Precision cancer medicine seeks to target the underlying genetic alterations of cancer; however, it has been challenging to use genetic profiles of individual patients in identifying the most appropriate anti-cancer drugs. This spurred the development of patient avatars; for example, patient-derived xenografts (PDXs) established in mice and used for drug exposure studies. However, PDXs are associated with high cost, long development time and low efficiency of engraftment. Herein we explored the use of microfluidic devices or microchambers as simple and low-cost means of maintaining bladder cancer cells over extended periods of times in order to study patterns of drug responsiveness and resistance. When placed into 75 µm tall microfluidic chambers, cancer cells grew as ellipsoids reaching millimeter-scale dimeters over the course of 30 days in culture. We cultured three PDX and three clinical patient specimens with 100% success rate. The turn-around time for a typical efficacy study using microchambers was less than 10 days. Importantly, PDX-derived ellipsoids in microchambers retained patterns of drug responsiveness and resistance observed in PDX mice and also exhibited *in vivo*-like heterogeneity of tumor responses. Overall, this study establishes microfluidic cultures of difficult-to-maintain primary cancer cells as a useful tool for precision cancer medicine.

## Introduction

Cancer remains a significant source of morbidity and mortality worldwide^1^. Traditionally, cancer treatment is designed based on the anatomical location and stage without considering the underlying cancer and individual patient variations. For example, all stage IV non-small cell lung cancers (NSCLC) are treated with platinum-based doublets while metastatic bladder cancer is usually treated with GC (gemcitabine and cisplatin/carboplatin) or MVAC (methotrexate, vinblastine, doxorubicin/adriamycin and cisplatin)^2^. Recent developments and clinical applications in “-omics” technologies brought forth the concept of precision cancer medicine which aims to individualize therapy based on the underlying molecular characteristics of cancer in specific patients. With the advance in omics technologies, a seemingly “uniform” cancer may be classified into several sub-groups and treated based on the underlying molecular and genomic alterations. For example, lung adenocarcinoma can be classified and treated based on EGFR activating mutation, EML4-ALK translocation and other genetic alterations. In fact, targeted therapy matched against these genetic alterations achieves a response rate of over 70%^3–5^, compared to less than 30% with non-specific platinum-based doublet chemotherapy^2^. Another success story for the field of precision cancer medicine is targeting of B-RAF mutation in melanoma^6,7^.

While these success stories are encouraging, the vast majority of cancer patients continue to be treated using “one-formula-fits-all” approach. A major reason for this is that each cancer harbors multiple genetic alterations, from a few in pediatric cancers to dozens and hundreds in adult cancers^8^, and computational biology tools are not able to distinguish the driver mutations from passenger mutations that have little impact on cancer function. Even in those patients whose cancers respond to targeted therapy, secondary resistance commonly develops within a few months. Biopsies of the resistant tumors have been used to study resistance mechanisms; however, small numbers of cells and impracticality of serial biopsies hinder the study of secondary resistance.

To address the unmet needs of precision cancer medicine, various cancer models have been established. One of the most commonly used *in vivo* models is patient derived xenografts (PDXs)^9,10^ which are developed from uncultured patient cancer cells. Human cancer cells have been engrafted into immunodeficient mice to create PDX models of pancreatic cancer, hepatocellular carcinoma, breast cancer, prostate cancer and many other cancer types^11–14^. Our team has a long-standing interest in bladder cancer and has recently reported the development of 22 PDX mice that could be used for drug screening purposes^15^. While extremely valuable, PDX models have disadvantages, such as long engraftment time (up to 6 months), low engraftment rate and high cost.

To address the drawbacks of *in vivo* models, several *in vitro* models have been developed with the goal to maintain phenotype of primary cancer cells long enough to evaluate cancer drug responsiveness and resistance^16–20^. These *in vitro* cultures aim to recapitulate aspects of tumor microenvironment including three dimensionality, heterotypic interactions and mechanical properties. 3D or spheroid cultures have proven particularly popular for drug screening and cancer biology studies^21–24^. Methods for forming spheroids have included hanging drops^25^, arrays of pyramidal wells^26^, microfluidic hydrodynamic traps^27^ and gel embedding^28^. However, there remains a need for new methods to maintain primary cancer cells *in vitro*.

**Figure 1:**
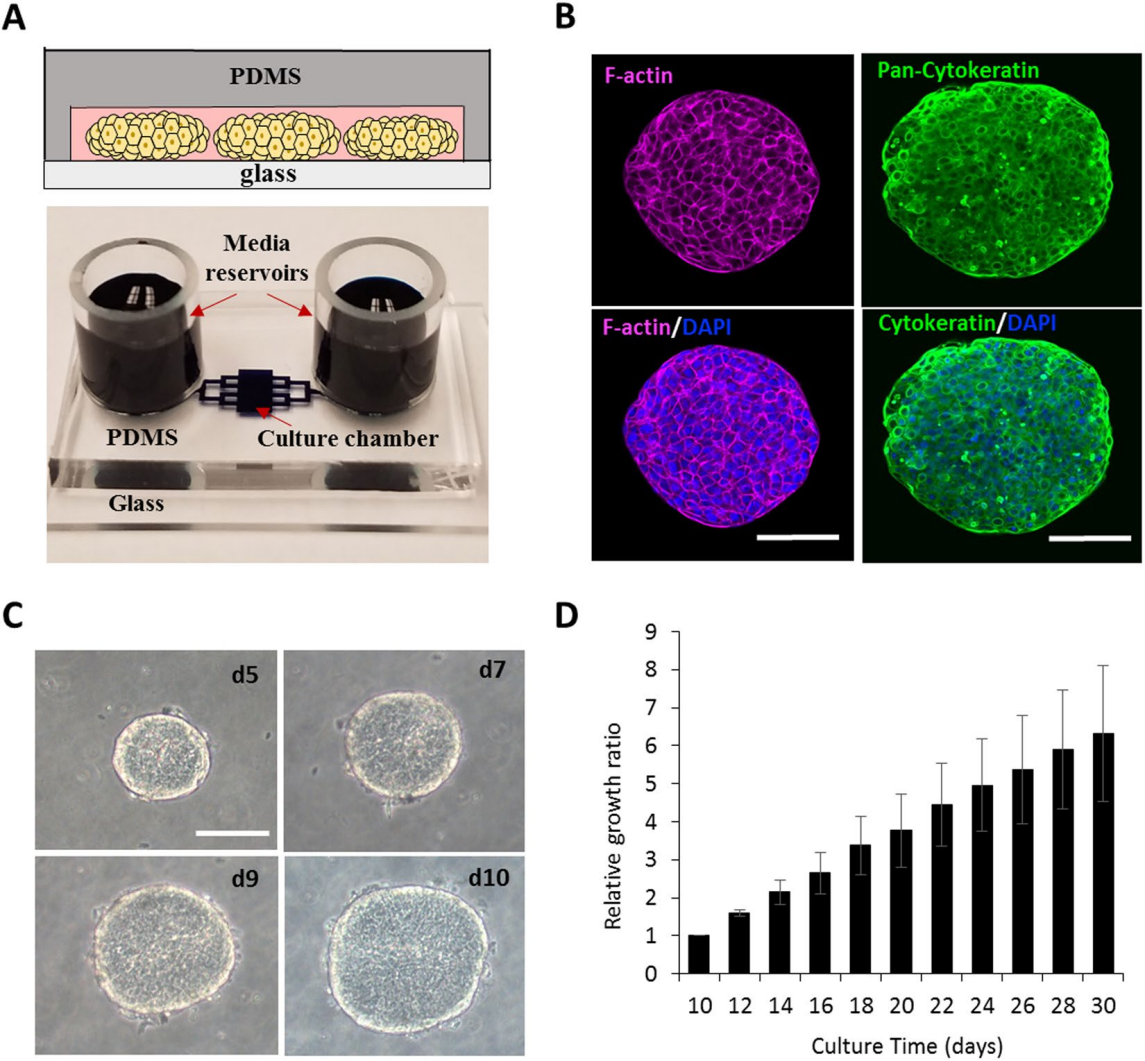
Bladder cancer cultures in microfluidic devices. (**A**) Top: depiction of ellipsoid cancer constructs in microchambers. Bottom: an image of a representative microchamber. (**B)** F-actin and cytokeratin staining demonstrates that bladder cancer ellipsoids retain epithelial phenotype after 10 days of culture in microchambers. Nuclei are stained with DAPI (Blue). **(C)** Bright field images showing growth of cancer ellipsoids in microchambers. **(D)** Growth of ellipsoids from BL0269 over 30 days in microchambers. Scale bars 100 μm.

Our team has recently described the use of pump-less microfluidic channels or microchambers for culturing primary hepatocytes and stem cells^29,30^. Here we investigated utility of microchambers for long-term maintenance of primary cancer cells which represents an important unmet need for the field of precision cancer medicine. As shown in Fig. 1A, our devices were simple microfluidic chambers with cloning cylinders serving as media reservoirs. Because the roof of microchambers was composed of oxygen-permeable silicone rubber, cancer constructs remained uniformly oxygenated and uniformly epithelial even when reaching millimeter lateral dimensions over 30 days in culture. Successful cultivation of bladder cancer cells from PDX mice as well as from patient cancer specimens was demonstrated. Beyond long term maintenance, we demonstrated that microchamber cultures from specific PDX models retained patterns of drug responsiveness and resistance observed in mice. This proof of concept study moves us closer to a microfluidics-based platform for long-term cultivation of cancer cells and screening of anti-cancer drugs.

## Results and Discussion

### Spatial confinement of bladder cancer spheroids in microchambers

We have previously observed that difficult-to-culture primary epithelial cells (hepatocytes) were maintained in microfluidic devices without perfusion for up to three weeks with minimal loss of phenotype or function^30^. Herein we wanted to explore the use of microchambers for cultivation of cancer cells. A typical microchamber used in this study is shown in Fig. 1A, with dimensions provided in Figure S1. Soft lithography was used to pattern transport channels and a cell culture chamber in polydimethyl siloxane (PDMS) – gas permeable silicone rubber. This PDMS top was reversibly bonded onto a glass coverslip and was outfitted with cloning cylinders serving as media reservoirs. Much like a regular culture dish, media exchange was accomplished by aspirating and refilling the reservoirs.

Our initial efforts to culture single cell suspension of bladder cancer cells were only moderately successful. To enhance survival and maintenance of cancer cells, we explored a strategy of forming multi-cellular clumps prior to seeding into microchambers^16^. Cancer tissue was carefully dissociated into cell clusters using collagenase type-I solution and separated using 40-µm cell strainer and 180-µm nylon mesh (Figure S2). Cell clusters captured in the strainer were cultured in 12-well plates (see Methods section for details) and, after 24 h, were seeded into microchambers. Typically, ~1 cm^3^ piece of tumor tissue yielded enough cell clumps to seed ~20 microchambers (1–20 clumps per device).

First, we characterized growth for bladder cancer cells in microchambers and Matrigel cultures. For these comparison studies, we used bladder cancer PDX BL0269 - developed from an invasive (advanced) bladder cancer^15^. As highlighted by a cartoon in Fig. 1A, the constructs growing in microchambers resembled ellipsoids and not spheroids, with z-axis defined by the 75 µm height of the microchamber. Growth was evaluated by measuring changes in volume using bright-field microscope and image analysis. Figure S3 highlights similarities in growth for both systems, suggesting that our microchamber cultures were comparable to the established Matrigel-based culture system. 3D bladder cancer constructs growing in microchambers stained strongly for cytokeratin and exhibited localization of F -actin to cell-cell junctions, suggesting maintenance of epithelial phenotype in (Fig. 1B). In addition to BL0269, we demonstrated maintenance of cells from BL0440 and BL0382 PDX mice (Figure S4). These three PDX models differed in patient age, cancer stage, drug resistance profile and prior exposure to chemotherapy^15^, suggesting that microchamber ellipsoid cultures were of general utility for maintenance of bladder cancer cells.

We should note that floating spheroids were also utilized as controls in our early experiments. However, we found this culture format to be suboptimal for monitoring changes in spheroid size over time and have focused on Matrigel-embedment of spheroids as a culture method to compare against our microfluidic cultures.

### Cancer ellipsoids may be cultured in microchambers without passaging for over 30 days

**Figure 2:**
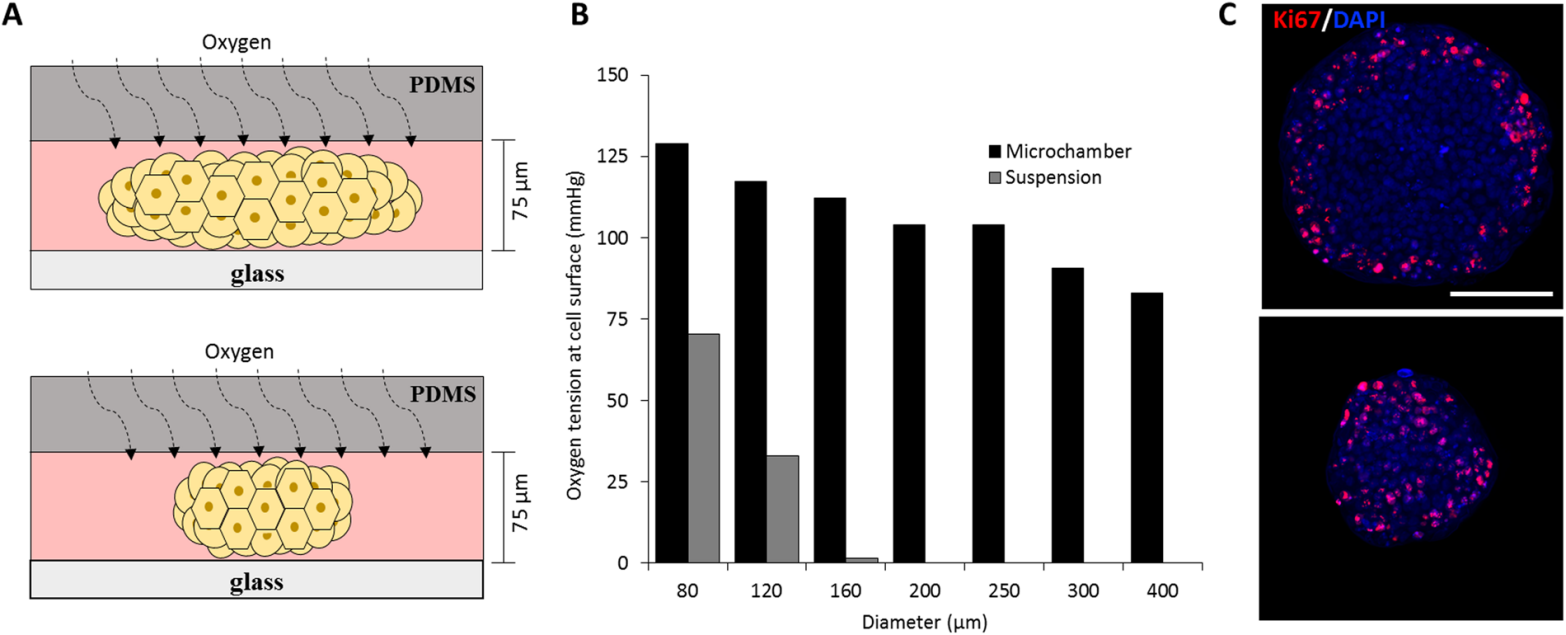
Cancer dormancy in microchambers. **(A)** Cartoon describing that oxygenation of ellipsoids is uniform regardless of xy dimensions. **(B)** Numerical simulation of oxygen tension at the center of cancer ellipsoids with various dimeters (xy plane) in microchambers vs. cancer spheroids in suspension culture following 12 hr of incubation. **(C)** Ki67 staining profile for large and small ellipsoids cultured for 16 days in microchambers. Scale bars 100 μm.

Tumors can grow in mice over a period of several weeks during which responsiveness and resistance to anti-cancer drugs may be studied. On the other hand, cancer spheroids cultured in suspension or in Matrigel slow down growth after two weeks in culture and develop necrotic cores when diameters exceed 400 µm^16,21^. While suitable for some drug screening applications, this timeframe may be insufficient for studies focusing on how anti-cancer drug resistance develops and whether salvage drug or drug combinations can overcome resistance. Unlike standard spheroid culture methods, the growth rate of cancer constructs in microchambers remained unchanged over 30 days of culture and there was no necrosis in the middle of ellipsoid tumor mass (Fig. 1C,D). The lack of necrotic core may be explained by the ellipsoid geometry of cancer constructs that allows for sufficient nutrient delivery and by the fact that the roof of the microchamber is composed of silicone rubber – an oxygen permeable material. The latter property of our culture system ensures uniform oxygen delivery regardless of the lateral dimensions, as highlighted by cartoon in Fig. 2A. Modeling of oxygen levels in the core of 400 µm diameter spheroids in suspension culture vs. ellipsoids in microchambers highlights the differences in oxygen tension between the two systems (see Fig. 2B and supplemental materials for details of COMSOL modeling). Interestingly, as shown in Fig. 2C, we observed differences in localization of Ki67-expressing proliferating cells depending on the size of ellipsoids. While proliferating cells were uniformly distributed throughout smaller ellipsoids (<150 µm diameter), these cells were confined to the periphery of larger constructs. A number of previous reports suggested hypoxia as the possible inducer of dormancy in cells located at the core of cancer spheroids^16,21,31,32^. Given sufficient oxygen delivery, differences in location of dormant and proliferating cells within ellipsoids of different sizes may be attributed to other, yet to be determined mechanisms.

### Drug responses of cancer ellipsoids parallel those observed in PDX mice

An important goal of this study was to compare drug responses of bladder PDX models *in vivo* and in microchamber cultures. We chose BL0269 PDX to carry out this comparison because this bladder cancer model had an interesting pattern of drug responsiveness^15^. It was relatively resistant to cisplatin and gemcitabine, two chemotherapy drugs commonly used for treatment of bladder cancer. However, BL0269 was found to harbor a PI3K H1047R activation mutation and was sensitive to a targeted therapeutic agent GDC-0941 – a small molecule inhibitor of PI3K.

**Figure 3:**
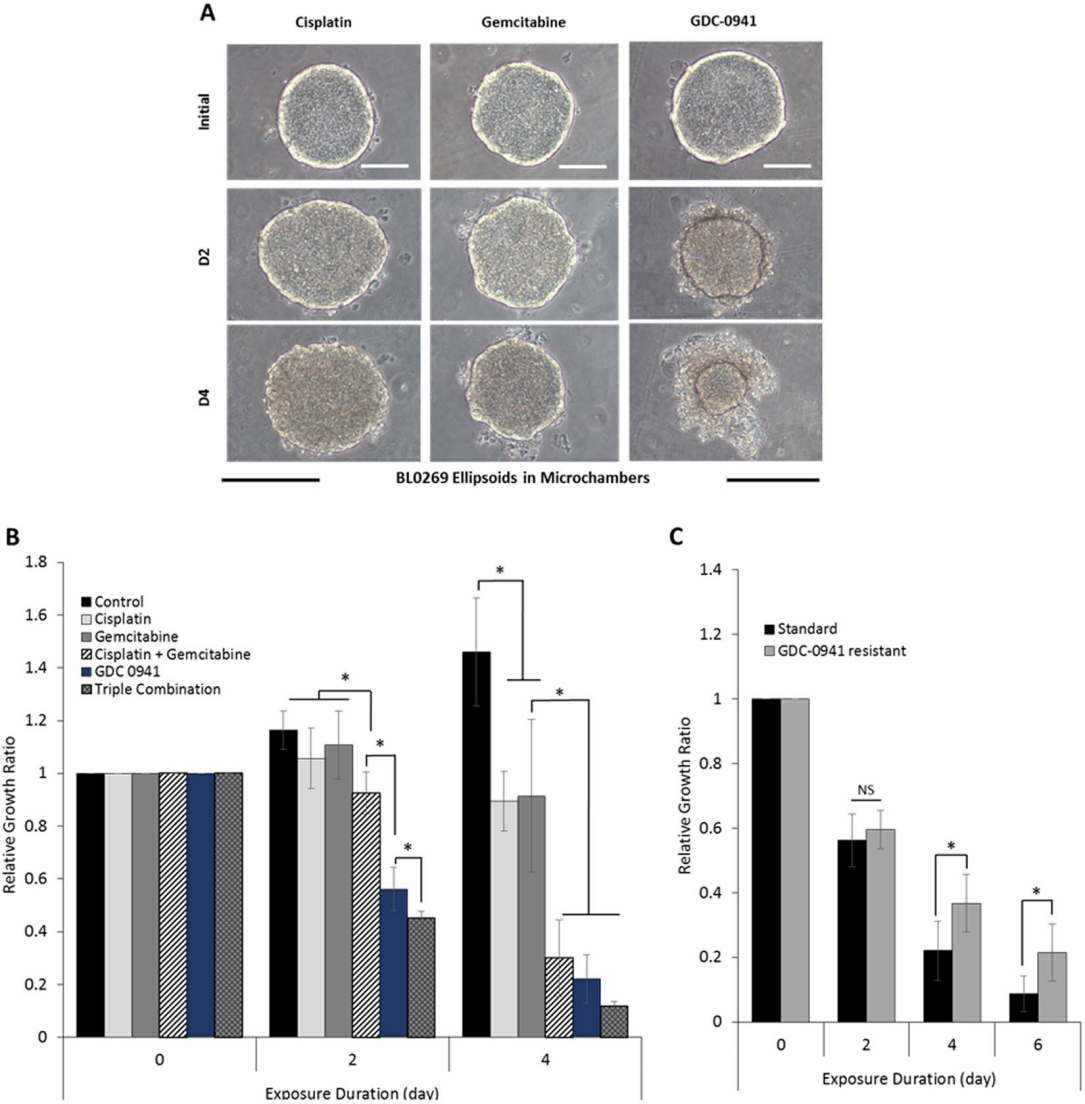
Drug responses of PDX-derived ellipsoids cultured microchambers. **(A)** Bright field images of BL0269 ellipsoids in microchambers treated with cisplatin (10 μM), gemcitabine (10 μM) or GDC-0941(1 μM). Cancer ellipsoids were cultured for 10 days prior to drug exposure. **(B)** Response of BL0269 ellipsoids to cisplatin (10 μM), gemcitabine (10 μM), cisplatin & gemcitabine (10 μM each), GDC-0941(1 μM) or triple combination. Untreated cancer ellipsoids served as control. Data represent the average of relative growth ratio of 6 biological samples per group ± SD; *p < *0.01*. **(C)** Responses of standard (GDC-0941 sensitive) and GDC-0941 resistant ellipsoids to 1 μM GDC-0941. Data represent the average of relative growth ratio of 7 biological samples per group ± SD; NS = non-significant; *p < *0.01*. Scale bars 100 μm.

We cultured BL0269 cells in microchambers for 10 days to allow ellipsoids to exceed 100 µm diameters and then challenged cultures with anti-cancer drugs for the additional 6 days. As seen from Fig. 3(A and B), BL0269 ellipsoids were almost unchanged after 48 h exposure to 10 µM cisplatin or 10 µM gemcitabine, but shrunk to half the size in response to 1 µM GDC-0941. The concentrations were chosen based on reports of efficacy of these drugs both *in vivo* and *in vitro* ^33–38^.

As seen from Fig. 3B, the pattern of responsiveness and resistance in microchamber cultures was very similar to our previous observations with PDX in NSG mice^15^. BL0269 was relatively resistant to cisplatin and gemcitabine single agents. The cisplatin/gemcitabine combination was slightly more effective than single agents. Because this tumor culture harbors a PI3K activation mutation, it was very sensitive to GDC-0941. The combination of three drugs was more effective than GDC-0941.

Another important experiment was carried out with BL0269 tumors that were resistant to GDC-0941. We noticed that, while daily treatment of PDX BL0269 mice with GDC-0941 caused tumor size to decrease initially, tumors became resistant over subsequent 20 days of treatment and began to increase in volume (unpublished data). These resistant BL0269 tumors were excised from mice, digested and cultured in our microchambers. The data comparing responses of GDC-0941 sensitive and resistant bladder cancers in microchambers is presented in Fig. 3C. These data show that, as expected, a decrease in size of sensitive BL0269 tumors was significantly greater than in resistant tumors when exposed to GDC-0941 (Fig. 3C). However, even the so-called GDC-0941-resistant cell culture still responded to GDC-0941 treatment, suggesting that some GDC-0941-sensitive cells survived GDC-0941 treatment in mice and repopulated during the initial microchamber culture before GDC-0941 treatment.

It should be noted that while MTT assay and Live/Dead staining were attempted as a means of evaluating cytotoxicity, these methodologies proved suboptimal for our purposes. MTT assay was challenging to normalize to the actual number of cells being exposed to drugs while dyes used for Live/Dead staining had limited permeability into ellipsoids being culture in our device. Therefore, susceptibility to drugs was assessed by measuring ellipsoid diameters in the x-y plane and monitoring change in ellipsoid volume over time. The diameter in z-axis was assumed to be equal to the height of the cell culture chamber – 75 µm. Changes in tumor volume are commonly used for both *in vitro* and *in vivo* assessment of responses to chemotherapy^16,32^.

### Cancer relapse in microchambers

Multiple studies have highlighted the importance of investigating relapse and heterogeneity of drug responses within cancer tissue^39,40^. A cancer model most commonly used for drug screening involves imbedding spheroids into Matrigel. This system helps fix spheroids spatially within the culture dish and allows one to characterize the growth of the same set of spheroids in response to anti-cancer drugs. While useful, such an approach has limitations. First, it is difficult to wash out dead cells from spheroids imbedded in Matrigel, meaning that the response to drugs is gauged by the rate of growth, the more aggressive the drug the smaller the rate. This makes it difficult to study slow growing cancers, including a number of primary cancers. Second, because dead cells remain associated with spheroids inside Matrigel, it is difficult to model relapse – a process where tumor grows back after withdrawal of anti-cancer drugs.

**Figure 4:**
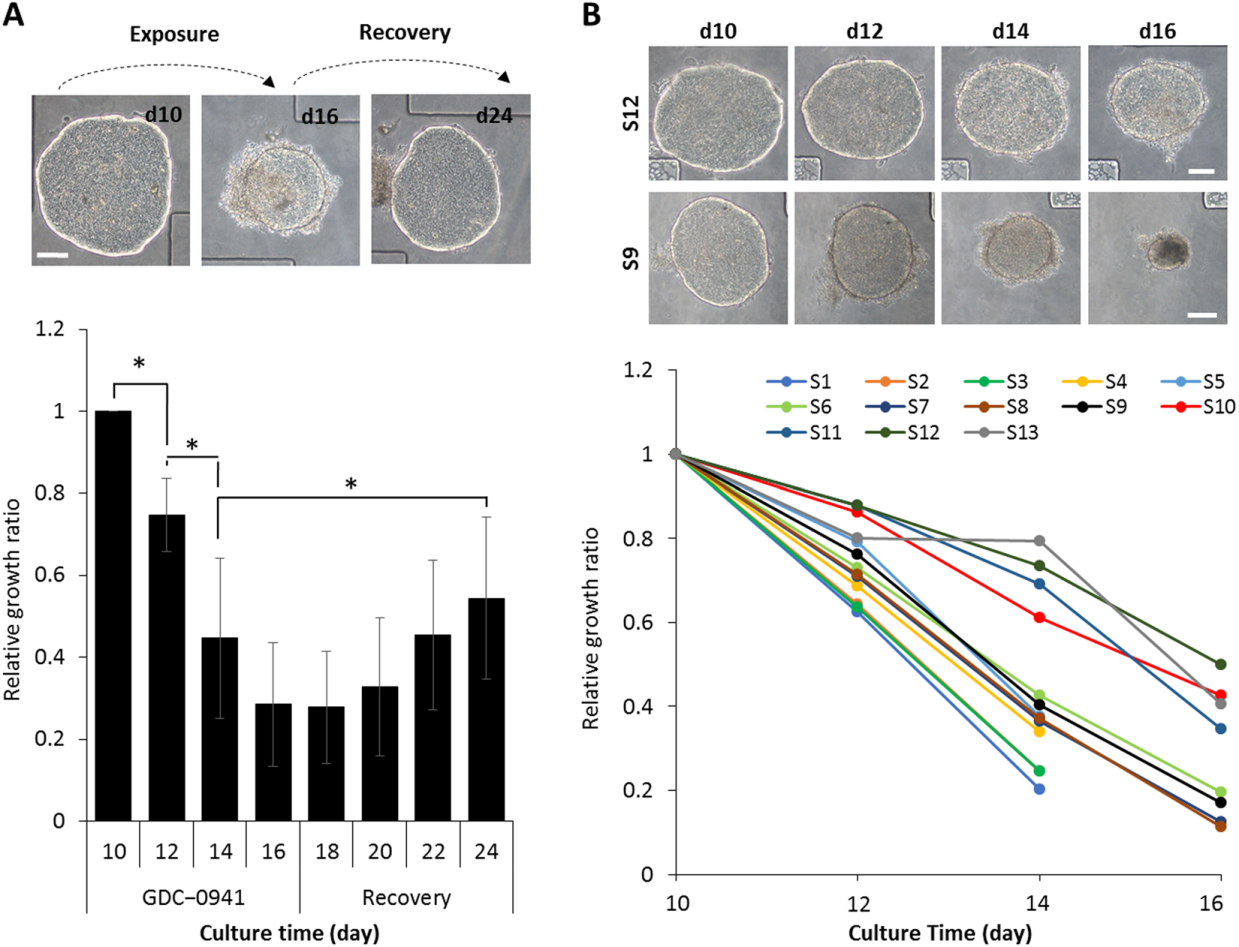
Tumor relapse and heterogeneity studies in microchambers. **(A)** Brightfield images and growth curve of BL0269 ellipsoids in microchambers treated with GDC-0941 from day 10 to 16 followed by eight days of drug withdrawal. Data represent the average of relative growth ratio of 7 biological samples per group ± SD; *p < *0.01*. Scale bars 100 μm. **(B)** Heterogeneity in responses of BL0269 ellipsoids to 1 μM GDC-0941. Top: images of resistant (S12) and sensitive (S9) ellipsoids (scale bars 100 μm). Drug treatment began after 10 days of culture and continued for 6 days. Bottom: changes in xy dimensions of 13 ellipsoids from the same BL0269 PDX mouse in response to 1 µM GDC-0941.

In contrast, ellipsoids in microchambers shrink and expand easily in response to presence or absence of anti-cancer drugs, making this system well suited for drug response and relapse studies. This point is illustrated by images in Fig. 4A which shows changes in BL0269 ellipsoid size in response to introduction and withdrawal of PIK3 inhibitor (GDC-0941). The BL0269 ellipsoids were first exposed to GDC-0941 for six days, followed by eight days of recovery period in which they were bathed in standard culture media. As shown in Fig. 4A, although initial drug treatment significantly shrunk ellipsoids, cancer cells repopulated the clumps after drug withdrawal.

In a clinical setting, cancer cells collected from the same tumor frequently exhibit heterogeneity in drug susceptibility. Analysis of 13 ellipsoids derived from the same PDX tissue and cultured in microchambers during exposure to GDC-0941 revealed considerable heterogeneity (Fig. 4B). Some clumps responded and disappeared after 14 days of treatment while other cancer constructs shrunk only to half the size during the same time. The differences and heterogeneity in responses of 3D constructs obtained from the same cancer tissue points to the possibility of investigating tumor heterogeneity using our culture system.

### Culturing primary bladder cancer cells in microchambers

**Figure 5:**
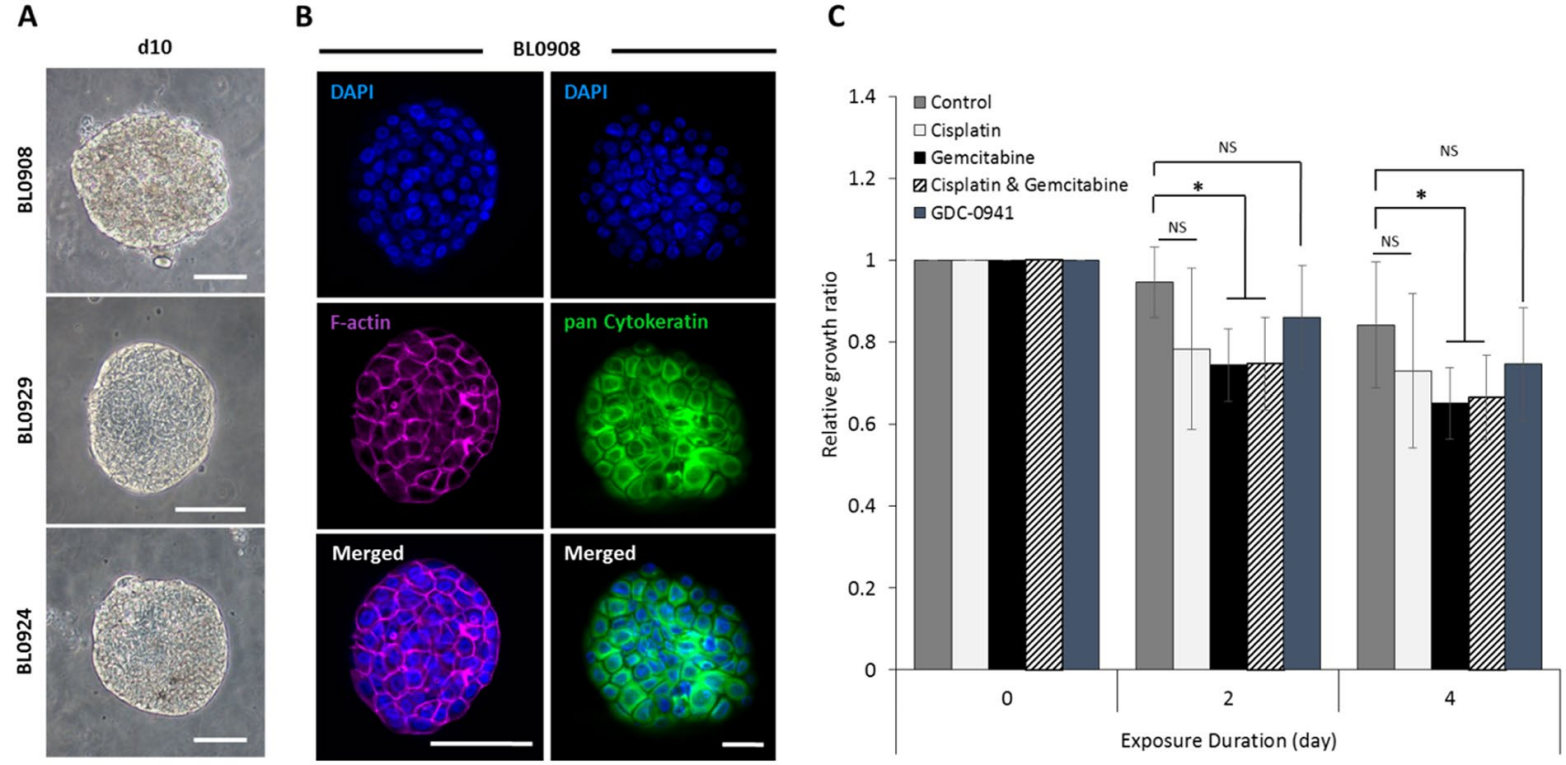
Phenotype and drug responses of primary bladder cancer cells in microchambers. (**A**) Representative images of ellipsoids from 3 different patients at day 10 in culture. (**B**) F-actin and cytokeratin staining in a representative primary cancer ellipsoid (BL0908) at day10 in microchambers. Nuclei are stained with DAPI (Blue). (**C**) Responses of BL0924 ellipsoids to cisplatin, gemcitabine, cisplatin & gemcitabine and GDC-0941. Untreated BL0924 served as control. Data represent the average of 7 biological samples per group ± SD; *p < *0.01*, NS = non-significant. Scale bars 100 μm.

Our ultimate goal is to establish primary cancer cell cultures for drug screening and tumor heterogeneity studies. As a step towards this goal, we carried out experiments with biopsies from three different bladder cancer patients – de-identified as BL0924, BL0929 and BL0908. Cancer tissue was dissociated into multi-cellular clumps, followed by seeding into microchambers as discussed earlier in the paper. Figure 5A shows representative ellipsoids from all three patients after 10 days of culture in microchambers. Retention of epithelial phenotype was confirmed by immunostaining which revealed strong expression of pan-cytokeratin and localization of F-actin cell-cell junctions (Fig. 5B). Following ten days of culture, BL0924 ellipsoids were exposed to gemcitabine, cisplatin and/or GDC-0941. As shown in Fig. 5C, only gemcitabine had a statistically significant effect on BL0924 ellipsoids compared to the control group. Neither cisplatin alone or in combination with gemcitabine was more effective than gemcitabine alone. Interestingly, unlike BL0269 PDX cells discussed earlier, BL0924 ellipsoids did not respond to GDC-0941 – suggesting that these cancer cells do not rely on the PI3K pathway for survival.

## Conclusions

In this study, we report for the first-time the culture of PDX-derived and primary bladder cancer cells in microfluidic devices. Bladder cancer cells formed ellipsoid-like constructs that exhibited growth and well-differentiated epithelial phenotype over the course of 30 days inside microchambers. A significant emphasis of the study was on demonstrating that drug response patterns of bladder cancer ellipsoids in microchambers paralleled those of matched PDXs in mice. Testing of BL0269 PDX cells *in vitro* revealed similar responsiveness to a PI3K inhibitor drug and resistance to chemotherapeutics, cisplatin and gemcitabine, as observed *in vivo*. Furthermore, PI3K-resistant subclones identified in PDX mice remained relatively resistant in microfluidic chambers.

Our method of 3D cancer cell culture has several advantages over other 3D culture approaches reported to date. 3D cell constructs cultured in our devices reach millimeter-scale xy dimensions without developing necrotic core – an issue that plagues standard cancer spheroid cultures^21,41^. This is due to the fact that oxygenation is uniform regardless of the tumor size in our microfluidic devices, and that ellipsoids receive sufficient nutrient levels.

3D constructs in our system are lodged between the floor and the roof of microchambers and are bathed in media. This ensures that dead cells are removed and tumors shrink upon drug exposure. In contrast, a standard way of embedding spheroids in Matrigel does not allow for removal of dead cells and therefore drug effects are gauged by the lack of growth and may be confounded by the dead cells attached to the tumor mass. This standard approach makes it difficult to study drug responsiveness of slow growing tumors whereas our method of monitoring shrinkage (or increase) in tumor size is well suited for this.

Furthermore, we made an interesting observation that location of proliferating cells within tumors in microchambers varied based on the tumor size. Previous studies attributed the presence of proliferating cells at the tumor’s edge and dormant cells in the tumor’s core to gradients in oxygen^42,43]^. Our culture system provides uniform oxygenation of 3D cancer constructs regardless of lateral dimensions and points to alternative mechanisms for cancer dormancy.

The focus of this study was on demonstrating maintenance of primary cancer cells and retention of *in vivo*-like drug responses in simple microfluidic devices. The complexity of such devices may be enhanced in the future by placing multiple culture chambers on the same chip and by including drug reservoirs, gradient generators and valves to control sequence of delivery, temporal patterns and concentrations of drugs. Such a platform will be an important step towards personalizing cancer treatment.

## Materials and Methods

### Fabrication of microchambers

Microfluidic chambers were fabricated using basic soft lithography techniques described by us previously^30^. Briefly, a master mold was fabricated using a photomask (AutoCAD/Art Services) for pattering SU-8 (Microchem Corp) on a silicon wafer (University Wafer). Single layer of polydimethylsiloxane (PDMS; Dow Corning) was made by pouring a mixture of 1:10 ratio of the curing agent to base onto the master mold. After a 30-min degassing step, PDMS was baked at 70 °C for 80 min. Solidified PDMS layer was removed from the master mold using No.11 scalpel blade after which inlet and outlets were created using a metal puncher. A PDMS layer was treated with corona discharge instrument and then irreversibly bound onto a glass slide (75 mm × 25 mm). We found it important to deconstruct microchambers for higher quality of immunostaining. To achieve this, we devised a reversible PDMS to glass bonding strategy by replacing the oxygen plasma treatment with 20 min baking at 70 °C. Microchambers were filled with deionized (DI) water and kept at 48 h at 4 °C prior to cell culture. Glass cloning cylinders (10 mm, Fisher Scientific) served as media reservoirs and were attached onto inlet and outlet using PDMS mixture as glue (1:10 ratio of curing agent to base). Microfluidic device with dimensions of individual channels is shown in Figure S1.

### Preparation of cancer spheroids from PDX and patient tumors

Bladder cancer patient derived xenografts (PDXs) were established in mice as described by us previously^15^. All animal protocols were approved by the UC Davis Institutional Animal Care and Use Committee (IACUC, Protocol No. 17794) and The Jackson Laboratory (JAX) IACUC (Protocol No. 12027). Collection of cancer tumor tissues and clinical information from patients was approved by the UC Davis Institutional Review Board (Protocol No. 218204). Written informed consent was obtained from all patients providing specimens and all experiments were performed in accordance to relevant guidelines and regulations.

Figure S2 displays schematic illustration of steps involved in preparation and formation of cancer spheroids from bladder cancer specimens. Identical protocol was carried out for PDX and patient tumor tissues. Prior to enzymatic digestion, a solid tissue specimen was placed in a sterile 10 cm petri dish having HBSS (with Ca and Mg), and then fragmented into small pieces (~mm) using a sterile No. 11 scalpel blade. Small tissue fragments were then incubated with collagenase type I (Gibco) in 10 ml of HBSS (with Ca and Mg) with final concentration of 235 U/ml. Enzymatic digestion was carried out in a 37 °C bath/shaker (200 rpm) for 25 mins. To isolate cell clusters with sizes ranging from 40 μm to 180 μm, digested tissue fragments were first passed through a sterile 180 μm size nylon mesh (Milipore), then a 40 μm cell strainer (Fischer Scientific). Cell clusters remaining on the 40 μm cell strainer were washed once with fresh culture media and dispensed in a standard 6-well plate. Remaining tissue fragments on the 180 μm net filter were disaggregated and digested for additional 30 mins. Digestion and filtration steps were repeated up to three times to collect sufficient number of cell clusters. Following an overnight incubation, isolated cell clusters spontaneously began to form spherical structures as previously reported by Kondo *et al*. for colorectal cancer tissues^16^. To ensure completion of cell clusters transformation into spheroids, suspension culture in a 6-well plate continued for 48–72 h following digestion.

Cancer spheroids derived from bladder cancer PDXs were maintained in RPMI (Gibco) supplemented with 30% (vol/vol) FBS (Invitrogen) and 1% (vol/vol) penicillin-streptomycin (Invitrogen); while primary cancer spheroids were cultured in RPMI (Gibco) with B27 (50X, Life Technology) and 1% (vol/vol) penicillin-streptomycin (Invitrogen) supplemented with bFGF (20 ng/ml, Invitrogen) and EGF (20 ng/ml, Invitrogen)^44^.

### Cancer ellipsoid culture in microchambers

Microchambers were first sterilized by 30 min UV exposure in the tissue culture hood, followed by coating with 0.25 mg/mL of poly-L-lysine (sigma) solution diluted in DI water for 1.5 h at 37 °C. Devices were then flushed with PBS, followed by RPMI-based culture media. After multi-cellular clumps of cancer cells have formed in suspension culture, they were collected and re-suspended in <500 μl of culture media. Spheroids suspension in 100 µl was introduced at the inlet of the microchamber and negative pressure was applied at the outlet using a syringe vacuum. Following introduction of desired number of spheroids in the cell culture chamber, flow was stopped and inlet and out reservoirs were filled with 250 μl of culture media (see Fig. 1A for example of a microchamber). The difference in surface tension between inlet and outlet media reservoirs created a very slow back and forth flow (on the order of microliters per hour) that enhanced diffusion –dominated delivery of nutrients to cells. The flow pattern was described in greater detail elsewhere^45^. Dimensions of the microfluidic chamber are provided in Figure S1.

To determine growth dynamics of ellipsoids in microchambers, we measured radii in the x-y plane and assumed the radius in the z-direction to be 75 µm. Our seeding protocol was designed to lodge spheroids between the floor and the roof of the microfluidic chamber, justifying the assumption of spheroid height being equal to the height of the chamber. For growth dynamics study described in Fig. 1D we began monitoring ellipsoids on day 10 and continued until day 30. Therefore, xy dimensions of ellipsoids determined on day 10 of seeding were used to normalize measurements of ellipsoids on later days.

### Cancer spheroids culture in Matrigel

In some of the experiments cancer spheroids were embedded in Matrigel and maintained in standard 48-well plates using protocols described in the literature^46^. Briefly, tissue culture wells were incubated with ~80 μl of Matrigel (BD Biosciences) at 37 °C for 20 min. Following gelation, cancer spheroids suspended in culture media were mixed with fresh Matrigel at 2.5% final concentration and then dispensed on the Matrigel-coated well. Media was exchanged every 48 h by gently removing the media from the top and adding 250 μl fresh culture media with 2.5% Matrigel.

To determine growth dynamics, radius of spheroids was determined using microscopy at the initial time point and then was used for normalizing radius measurements on subsequent days. Matrigel embedded spheroids were assumed to grow uniformly in x-y-z direction.

### Evaluation of drug responses of cancer cell cultures in microchambers

Changes in cancer ellipsoids size in the xy-plane were used to determine drug response. Time-lapse bright field images were processed using ImageJ to calculate the changes in the area (xy-plane) occupied by each ellipsoid before and after drug treatments. Utilizing “Macros” plugin, the threshold and other settings were kept the same for all processed images. Area of each ellipsoid in the xy-plane after drug treatment was divided by its initial measurement for calculating the relative growth ratio as a means of determining drug response.

### Immunostaining

We modified a previously published protocol to achieve effective immunofluorescent staining of 3D cancer constructs^47^. Prior to fixation of cells, culture media was removed from microchambers and channels were washed twice with PBS. Ellipsoids in microfluidic devices were fixed and permeabilized simultaneously at 4 °C by exposure to 4% paraformaldehyde (Electron Microscopy Sciences) and 1% Triton X-100 (Invitrogen) in PBS for 3 h. Using washing solution (0.1% Triton in PBS), samples were flushed three times and then blocked overnight with 3% BSA and 0.1% Triton in PBS solution at 4 °C. Prior to antibody staining, a PDMS microchamber were removed from atop of the ellipsoids and replaced with a clean cloning cylinder (10 mm diameter) which served as solution reservoir. This step was critical to ensure successful penetration of antibodies to the center of compact cancer ellipsoids. Samples were then incubated for 48 h at 4 °C with primary antibodies diluted in incubation solution (PBS with 1% BSA and 0.1% Triton X-100). A list of primary antibodies and their dilution information are provided in Table S2. Samples were washed 5 times and then exposed to secondary antibodies diluted in incubation solution for 24 h at 4 °C. Excess secondary antibody solution was then removed by flushing with washing solution 5 times. Secondary antibodies used were: Alexa-546 donkey anti-rabbit (1:800) and Alexa-488 donkey anti-mouse (1:800). Cell nuclei and F-actin were stained by incubation with DAPI (1:900) and Alexa Fluor 647 Phalloidin (1:15, Cell Signaling) solution at room temperature for 1.5 h. DAPI and Phalloidin were both diluted in incubation solution. Afterwards samples were flushed 5 times with washing solution and incubated at 4 °C in PBS. Laser scanning confocal microscope (LSM700, Carl Zeiss, Jena, Germany) was used to image all samples.

### Statistical analysis

Student t-test was used for statistical analysis. Minimum four biological duplicates were used for each condition and standard deviations were presented as error bars. Number of biological duplicates and p value threshold used for each experiment is listed in the figure captions.

## Electronic supplementary material

Supplementary Information

Ki67 staining at different focal planes in smaller cancer ellipsoid
