# Supplementary material for: Microchamber Cultures of Bladder Cancer: A Platform for Characterizing Drug Responsiveness and Resistance in PDX and Primary Cancer Cells
Source: Sci Rep. 2017 Sep 25;7:12277. doi: 10.1038/s41598-017-12543-9 (PMC5612935; doi:10.1038/s41598-017-12543-9)
Supplement: Supplementary file 1 — Supplementary Information [file 41598_2017_12543_MOESM1_ESM.docx]

**SUPPORTING INFORMATION**

**Microchamber Cultures of Bladder Cancer: A Platform for Characterizing Drug Responsiveness and Resistance in PDX and Primary Cancer Cells**

Pantea Gheibi,^1^ Shuxiong Zeng,^2^ Kyung Jin Son,^1^ Tam Vu,^1,3^ Ai-Hong Ma,^2^ Marc A. Dall’Era,^4^ Stanley Alexander Yap,^4^ Ralph W. De Vere White,^4^ Chong-Xian Pan ^2,4 *^ and

Alexander Revzin ^1,5 *^

^1^ Department of Biomedical Engineering, University of California, Davis, CA 95616, USA

^2^ Department of Internal Medicine, Division of Hematology/Oncology, University of California Davis, Sacramento, CA 95817, USA

^3^ Division of Rheumatology, Allergy and Clinical Immunology, University of California, Davis, CA 95616, USA

^4^ Department of Urology, University of California Davis, Sacramento, CA 95817, USA

^5^ Department of Physiology and Biomedical Engineering, Mayo Clinic, Rochester, MN 55905, USA

^*^ Corresponding authors:

**SUPPLEMENTAL FIGURES AND CAPTIONS**


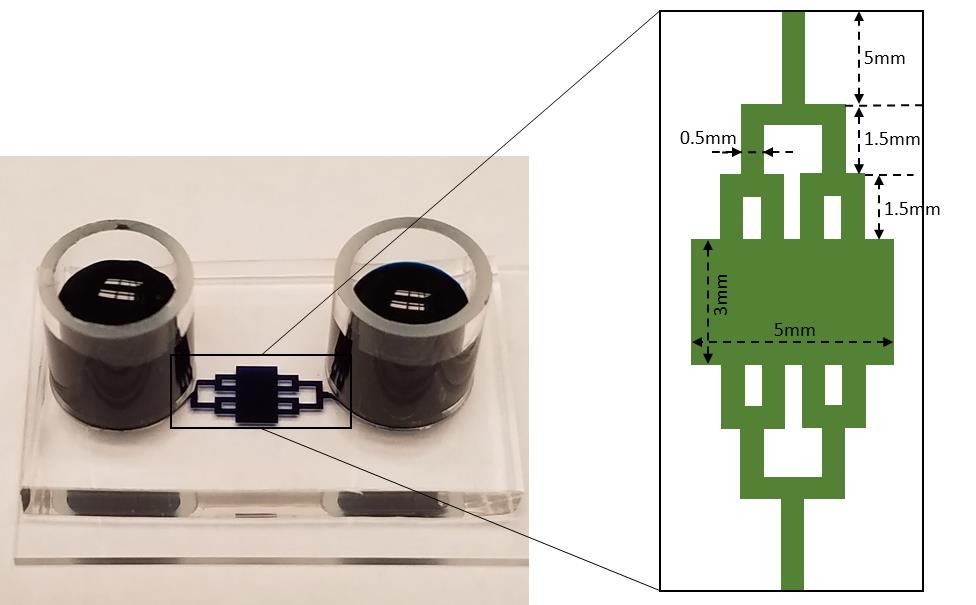


**Figure S1**. A microfluidic cell culture device with dimensions. Inset (schematic in green) shows dimensions of PDMS microfluidic layer. The height of all channels was 75 µm. Volume of each cloning cylinder serving as inlet and outlet reservoirs is 250 µL.

**
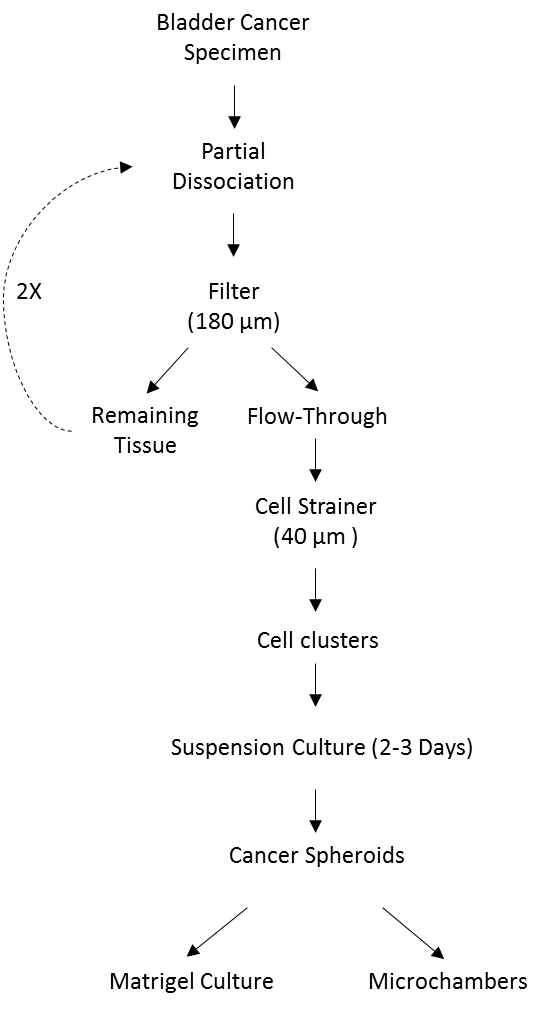
**

**Figure S2.** Schematic illustration of steps involved in preparation and formation of cancer spheroids Matrigel and microchamber cultures.


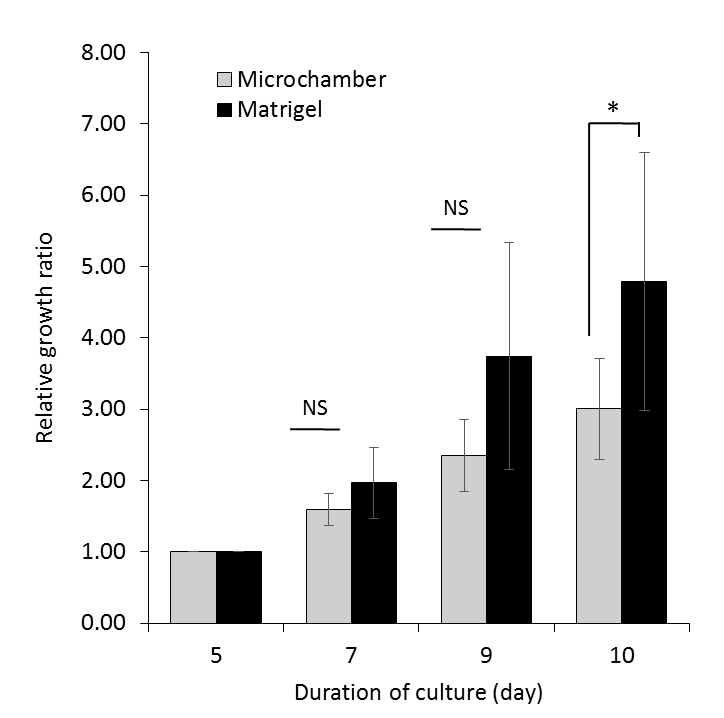


**Figure S3.** Relative growth ratio of BL0269 cancer spheroids embedded in matrigel and cancer ellipsoids in microchambers. Relative growth ratios were normalized to day 5 and represent the average of 5 biological samples ± SD; *p<0.05, NS=non-significant.


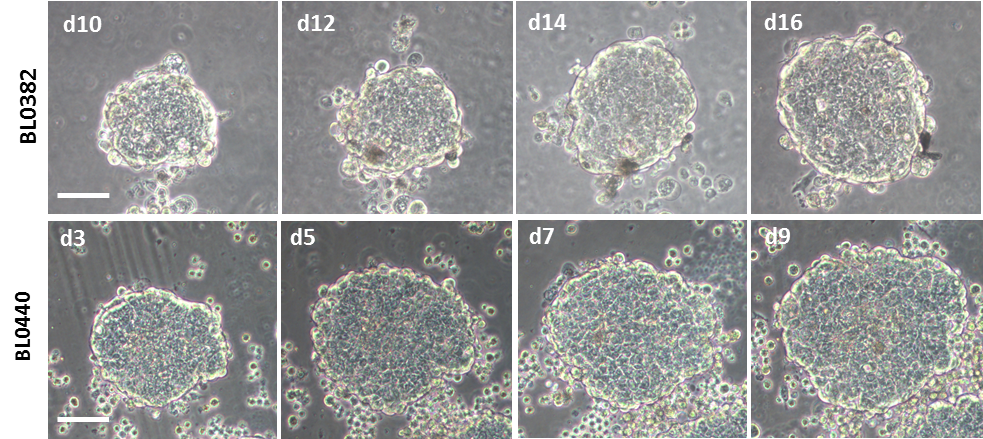


**Figure S4.** Brightfield images of ellipsoids from BL0382 and BL0440 cultured in microchambers.

**SUPPLEMENTAL MATERIALS AND METHODS**

**COMSOL numerical simulations.** Oxygen tension at the center of cancer ellipsoids cultured inside microchambers and cancer spheroids in standard culture system (suspension culture) were modeled using COMSOL Multiphysics 4.3 software (COMSOL Inc., Los Angeles, CA). We assumed that the following values remained constant for both culture settings: oxygen consumption rate and diffusion of oxygen inside cancer spheroids/ellipsoids. All parameters used for numerical simulations are provided in Supplementary Table 1.

1. **Oxygen tension levels at the center of cancer ellipsoids/spheroids**

Oxygen diffusion and consumption by cells was modeled using equation (1) for determining oxygen tension levels at the center of cancer ellipsoids/spheroids in both cell culture settings:

$\left( 1 \right) \frac{\partial c_{O_{2}}}{\partial t}=\nabla\cdot\left( D_{i}\nabla c_{O_{2}} \right)-u\cdot\nabla c_{O_{2}}+R_{i}$

In equation (1), $c_{O_{2}}$ is oxygen concentration (mol L^-1^), *u* is the flow velocity and *D_i_* is the diffusivity of oxygen (cm^2^ s^-1^) in cancer spheroids, medium and PDMS membrane. *R_i_* is the oxygen consumption of cells described by Michaelis-Menten kinetics as follows:

$${\left( 2 \right) R}_{i}= -\frac{V_{max}\rho_{cell}c_{O_{2}}}{K_{m}+c_{O_{2}}}$$

In equation (2), *V_max_* is the oxygen consumption rate per cell (nmol cell^-1^ s^-1^), and *K_m_* is the Michaelis-Menten constants (μM). The initial oxygen concentrations were set at 220 μM for cancer spheroids, 0 μM for medium, and 250 μM for PDMS membrane ^1^.

Following assumptions were made for simplification of modeling: 1) there exist a continuous pO_2_ and mass flux of oxygen at interfaces between cell and medium or medium and PDMS; 2) the pO_2_ on PDMS surfaces is constant (pO_2_ at PDMS surface = *P_g_* $\times$ *s_PDMS_* where *s_PDMS_* is oxygen solubility in PDMS); 3) glass layer at the bottom of microchamber and plastic bottom of the tissue culture plate are oxygen-impermeable.

**SUPPLEMENTARY TABLES**

**Table S1.** Parameters for oxygen tension modeling.

| **Parameters** | **Values** |
| --- | --- |
| Oxygen Consumption rate ($V_{max}$) | 0.0625$\times$10^-6^ (nmol s^-1^ cell^-1^) ^2^ |
| Diffusion Coefficient of Oxygen in PDMS | 7.88$\times$10^-5^ (cm^2^ s^-1^) ^1^ |
| Diffusion Coefficient of Oxygen in media | 3.0$\times$10^-9^ (m^2^ s^-1^) ^3^ |
| Diffusion Coefficient of Oxygen in cell | 2.0$\times$10^-9^ (m^2^ s^-1^) ^3^ |
| Oxygen Solubility in PDMS | 1.25 (mM atm^-1^) ^1^ |
| Oxygen Solubility in culture media | 0.22 (mM atm^-1^) ^1^ |
| Oxygen Solubility in cell | 1.049 (mM atm^-1^) ^1^ |
| Cell density inside tumor spheroids | 3.44$\times$10^-4^ (cells μm^-3^) |
| Michaelis constant ($K_{m}$) | 1 (μM) ^3^ |
| Media volume | 500 µL / device  12-well plate: 1 mL / well |

**Table S2.** Primary antibodies used for immunostaining

| **Antibody** | **Manufacturer** | **Host** | **Fluorescently labeled** | **Antibody dilution** |
| --- | --- | --- | --- | --- |
| Ki67 | Cell signaling | Rabbit | - | 1:250 |
| E-cadherin | BD | Mouse | - | 1:40 |
| Pan-Cytokeratin | eBioscience | Mouse | Alexa Fluor 488 | 1:80 |

**REFERENCE:**

1. Kim, M.C., Lam, R.H.W., Thorsen, T. & Asada, H.H. Mathematical analysis of oxygen transfer through polydimethylsiloxane membrane between double layers of cell culture channel and gas chamber in microfluidic oxygenator. *Microfluid Nanofluid* **15**, 285-296 (2013).

2. Wagner, B.A., Venkataraman, S. & Buettner, G.R. The rate of oxygen utilization by cells. *Free Radic Biol Med* **51**, 700-712 (2011).

3. Buchwald, P. FEM-based oxygen consumption and cell viability models for avascular pancreatic islets. *Theor Biol Med Model* **6**, 5 (2009).
