# Supplementary figures and images for: Microchamber Cultures of Bladder Cancer: A Platform for Characterizing Drug Responsiveness and Resistance in PDX and Primary Cancer Cells
Source: Sci Rep. 2017 Sep 25;7:12277. doi: 10.1038/s41598-017-12543-9 (PMC5612935; doi:10.1038/s41598-017-12543-9)

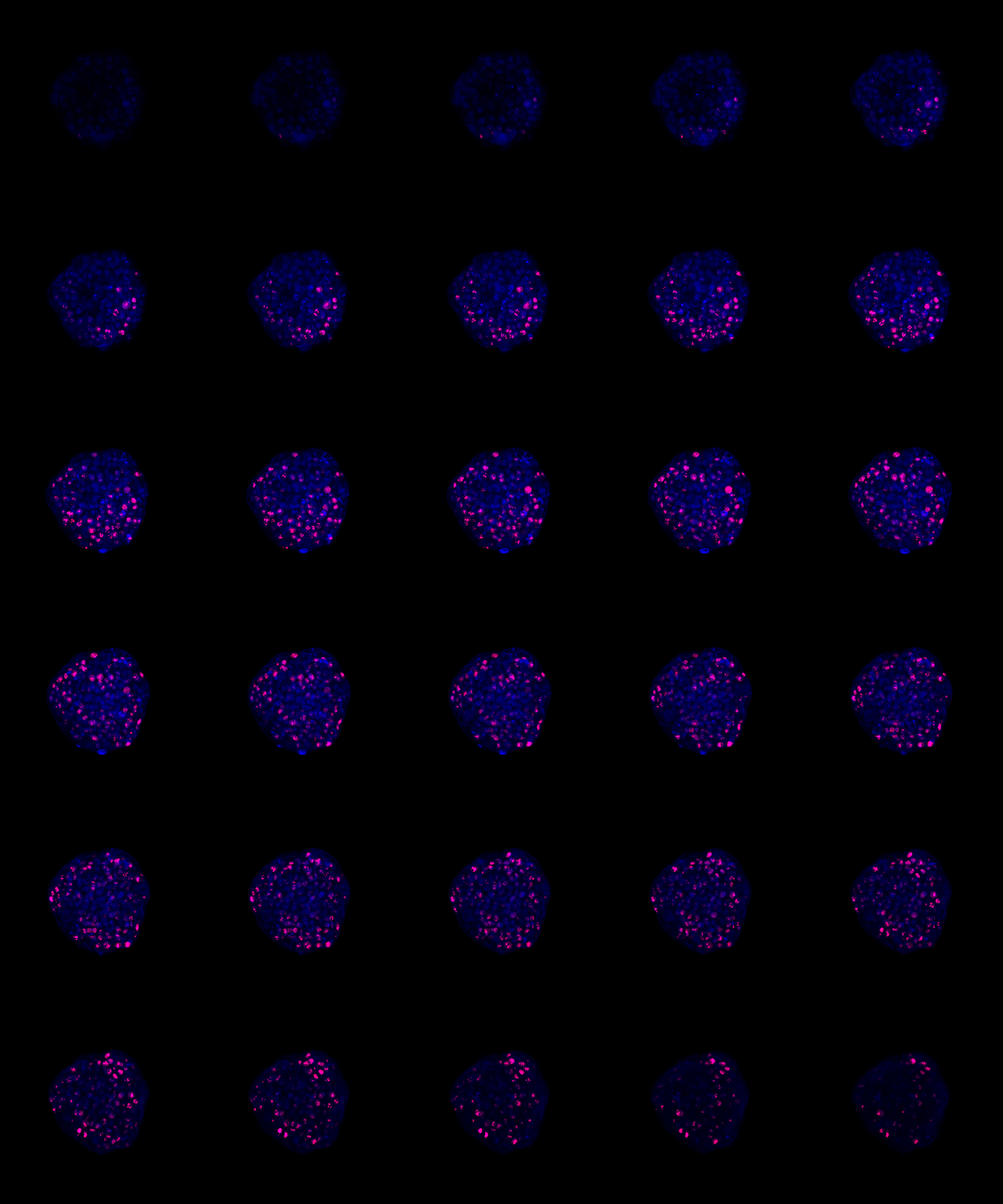

Supplement: Supplementary file 2 — Ki67 staining at different focal planes in smaller cancer ellipsoid [file 41598_2017_12543_MOESM2_ESM.jpg]
